# Anytoles

**Published:** 1898-06-18

**Authors:** 


### ANYTOLES.

A paper recently appeared in the *Deutsche Medicinische Wochenschrift* by Professor Loeffler, of the University of Greifswald, in regard to certain interesting oily bodies which have received the name of anytoles. In researches, which had been made as to the nature of icthyol, it was discovered that by the action of sulphuric acid upon various mineral oils and other hydrocarbons a new series of compounds could be formed which possessed the interesting property of rendering substances soluble in water which were not normally capable of solution in that medium. A series of comparative trials has ultimately led Helmers to the conclusion that hydrocarbons containing about 10 per cent. of sulphur chemically combined are the best. If these hydrocarbons are treated with concentrated sulphuric acid, then neutralised with ammonia, and the insoluble portion precipitated with alcohol, a product is obtained which possesses a great solvent action on bodies insoluble in water. This ammonia salt, formed from the sulphonated hydrocarbon rich in sulphur, is called by Helmers "anytin," and the compounds formed by it, and thus made soluble in water, are called "anytoles." The practical point, then, is that a whole series of preparations are now at our disposal in which such substances as phenol, cresol, guaiacol, &c., exist in a soluble form. The anytin itself, so far as its bactericidal action is concerned, seems to occupy about the same position as ichthyol, but the compounds possess the properties of the substances with which the anytin may be combined. What the future of these compounds may be it is impossible to say, but there seems before them a large field of usefulness.

